# Association between serum uric acid levels and dietary fiber intake in adults: the Korea national health and nutrition examination survey (KNHANES VII, 2016–2018)

**DOI:** 10.1186/s12986-024-00809-9

**Published:** 2024-06-10

**Authors:** Jinyoung Kim, Da Young Jung, Jin-Hee Lee, Mee Kyoung Kim, Hyuk-Sang Kwon, Hyeon Woo Yim, Su-Jin Moon

**Affiliations:** 1 Division of Endocrinology and Metabolism, Department of Internal Medicine, Yeouido St. Mary’s Hospital, The Catholic University of Korea, Seoul, Korea; 2 Department of Biostatistics, Clinical Research Coordinating Center, Catholic Medical Center, The Catholic University of Korea, Seoul, Korea; 3 Catholic Institute of Smart Healthcare Center, The Catholic University of Korea, Seoul, Republic of Korea; 4 Department of Preventive Medicine, The Catholic University of Korea, Seoul, Korea; 5 Division of Rheumatology, Department of Internal Medicine, Yeouido St. Mary’s Hospital, College of Medicine, The Catholic University of Korea, Seoul, Korea

**Keywords:** Dietary fibers, Uric acid, Nutrition survey

## Abstract

**Background:**

Hyperuricemia could be a risk for various chronic diseases, and it could be largely corrected by diet control. This study was a nationwide cross-sectional study to investigate the association between serum uric acid level and dietary fiber intake.

**Methods:**

This study analyzed data based on the Korean National Health and Nutrition Examination Survey conducted from 2016 to 2018. Adults over 20 years of age with normal renal function, defined as an estimated glomerular filtration rate (eGFR) over 30mL/min/1.73m^2^, were included. The criteria for hyperuricemia were ≥ 7 mg/dL in men and ≥ 6 mg/dL in women. Data regarding dietary intake were obtained using the 24-hour recall method.

**Results:**

A total of 15,278 subjects (6,455 males/8,823 females) were analyzed. The prevalence of hyperuricemia was 19.3% in men and 6.8% in women. There were significant, negative associations between serum uric acid and total fiber intake in both men and women. Consuming more than 27.9 g of dietary fiber in men and 20.7 g in women reduced the risk of hyperuricemia by approximately 30% with odds ratios of 0.72 (0.62–0.83) and 0.71 (0.56–0.88) in men and women, respectively. With regard to the risk reduction by the type of dietary fiber, cereal fiber was significantly identified in both men and women, while fruit fiber was only significant in men. In the subgroup analysis, this association remained significantly in young and metabolically healthy populations with normal weight.

**Conclusions:**

Dietary fiber intake was inversely associated with serum uric acid levels. This relationship was particularly significant in metabolically healthy young adults.

**Supplementary Information:**

The online version contains supplementary material available at 10.1186/s12986-024-00809-9.

## Background

The association between hyperuricemia and obesity, metabolic syndrome, and diabetes is well known [1, 2]. Hyperuricemia is also known to be an independent risk factor for metabolic syndrome and type 2 diabetes [1, 2]. High serum uric acid levels are also associated with other chronic disorders, such as gout [3] and impaired renal function [4]. Several studies have described a relationship between cardiovascular disease and hyperuricemia, including a correlation with mortality [5, 6]. Therefore, studying hyperuricemia is important to predict and prevent various chronic diseases and promote health [7].

Hyperuricemia can be largely corrected by lifestyle changes, and by diet control in particular [8]. One prior group found that the dietary factors associated with hyperuricemia include high intake of alcohol, sugary beverages, or red meat and low intake of vitamin C, folate, calcium, or fiber [9, 10]. The United States (US) National Health Survey and the China Adult Chronic Disease and Nutrition Surveillance both described how serum uric acid levels were inversely correlated with dietary fiber intake [11, 12].

Accordingly, we sought to confirm the relationship between dietary fiber intake and serum uric acid levels through analysis of the Korean National Health and Nutrition Examination Survey (KNHANES).

## Methods

### Study population

**Fig. 1:**
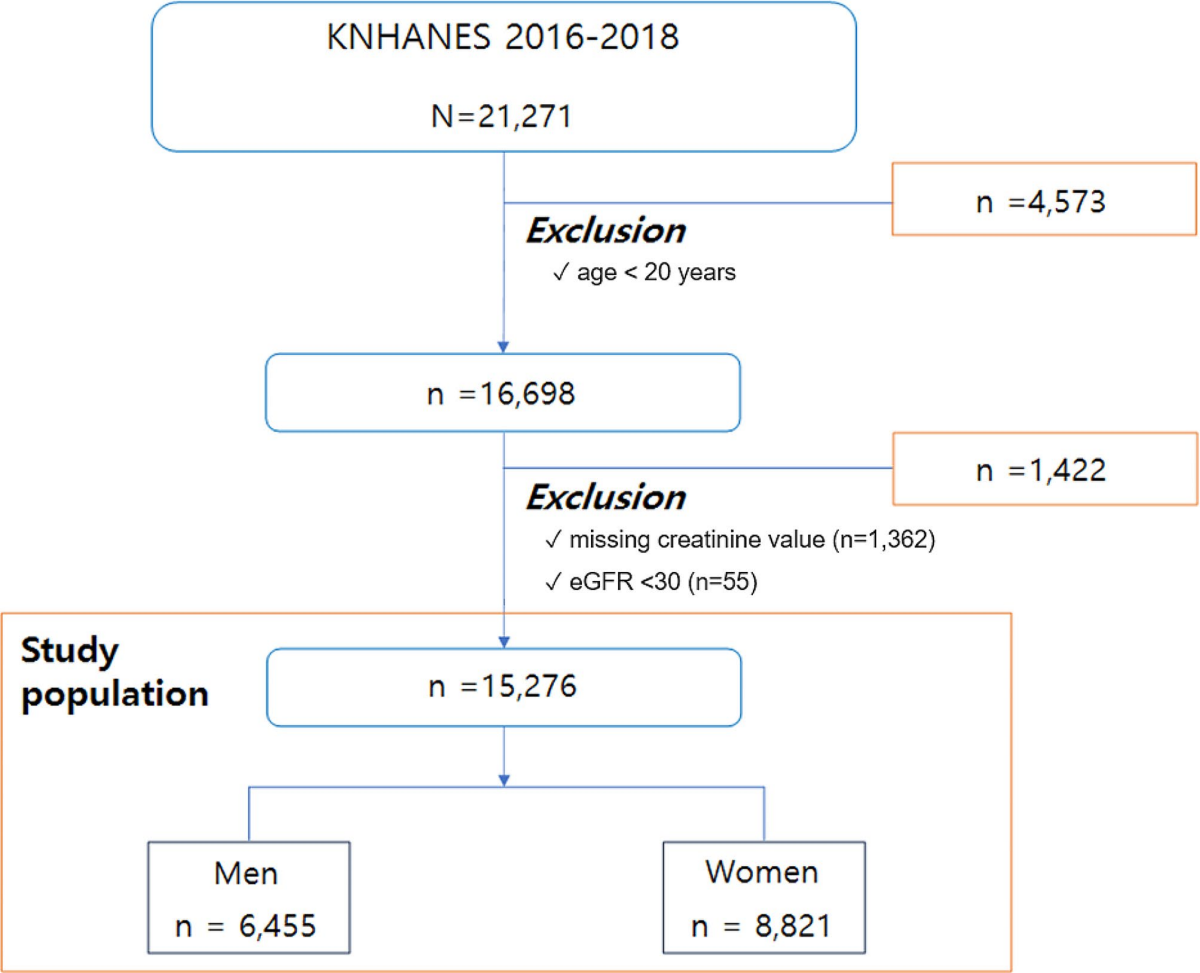
Study flowchart

This study analyzed data from the 7th Korean National Health and Nutrition Examination Survey (KNHANES) conducted from 2016 to 2018, which included serum uric acid levels. We screened adults older than 20 years. The association between hyperuricemia and chronic kidney disease may result from a decrease in the extracorporeal excretion of uric acid [13]. Therefore, we excluded patients with decreased renal function, defined as an estimated glomerular filtration rate (calculated with the Chronic Kidney Disease Epidemiology Collaboration (CKD-EPI) equation [14]) less than 30mL/min (Fig. 1). The study protocol was approved by the institutional review board of Yeouido St. Mary’s Hospital (SC21ZESI0114). Informed consent was waived due to the use of anonymous public data.

### Measurement and definition

In the physical examination for KNHANES, height and weight were measured using an automated body composition analyzer, and blood sampling for the biochemical study was performed after overnight fasting for more than 8 hours. Self-administered questionnaires were used for the medical history and lifestyle survey. Dietary intake was evaluated using the individual 24-hour recall method. The dietary fiber intake was calculated by applying the database of the ingredient amount and the nutritional component according to the food types [15].

For the evaluation of chronic diseases, the values ​​set in the KNHANES dataset were used. The chronic diseases were defined as follows: diabetes by a fasting blood sugar of ≥ 126 mg/dL, previous doctor’s diagnosis, or the use of oral hypoglycemic agents or insulin injections; hypertension by a systolic blood pressure ≥ 140mmHg or diastolic blood pressure ≥ 90mmHg, or the use of antihypertensive drugs; hyperlipidemia by a total cholesterol of ≥ 240 mg/dL or the use of cholesterol-lowering drugs. The lifestyle factors were defined as follows: smoking was defined by a prior history of smoking more than 5 packs (100 cigarettes) total and current smoking; alcohol use was defined by drinking alcohol more than once a month; moderate-intensity exercise was defined by activity that makes one slightly breathless or makes the heart beat slightly faster in leisure time (for example, jogging, weight training, golf, pilates, etc.).

Serum uric acid was measured using the colorimetry method using the Hitachi Automatic Analyzer 7600 − 210 (Hitachi / JAPAN). The cut-offs for hyperuricemia in males and females were set differently at 7 mg/dL and 6 mg/dL, respectively, based on previous studies conducted in the US and China.

### Statistical analysis

We used a complex sample design model for baseline characteristics. To compare the characteristics between groups, the t-test for continuous variables and Chi-square test for categorical variables were used. The correlation between the serum uric acid level and total dietary fiber intake was evaluated using linear regression. The receiver operating characteristic (ROC) curve was used to calculate the optimal cut-off dietary fiber level according to the hyperuricemia status. Univariate and multivariate logistic regression were performed according to the quartile of total dietary fiber. The same analysis method was additionally applied to subgroups according to the different fiber types and demographic characteristics of the study population. Statistical significance was calculated through complex sample analysis with demographic weights. A two-sided p value of less than 0.05 was considered statistically significant. Statistical analyses were performed using SAS version 9.3 (SAS Institute, Cary, NC, USA).

## Results

### Baseline characteristics of the study population

A total of 15,278 subjects (6,455 males/8,823 females) were analyzed. The prevalence of hyperuricemia was 19.3% in men and 6.8% in women. Baseline characteristics according to sex and serum uric acid status are included in Table 1. The hyperuricemia group generally weighed more, had higher liver enzyme levels, and had higher prevalence values of hypertension and hyperlipidemia than did the non-hyperuricemia group. Regarding lifestyle factors, drinking habits in men and current smoking status in women were significantly different between the groups.

**Table 1:** Baseline characteristics of the study population

mean ± SE	Men				Women			
	Total	Normal	Hyperuricemia	*P*	Total	Normal	Hyperuricemia	*P*
	*n* = 6,455	*n* = 5,212	*n* = 1,243		*n* = 8,823	*n* = 8,219	*n* = 604	
**Serum uric acid (mg/dL)**	5.94 ± 0.02	5.44 ± 0.02	7.84 ± 0.02	< 0.001	4.41 ± 0.01	4.25 ± 0.01	6.61 ± 0.03	< 0.001
**Age (years)**	46.75 ± 0.29	47.87 ± 0.31	42.46 ± 0.50	< 0.001	48.61 ± 0.28	48.21 ± 0.28	54.33 ± 0.92	< 0.001
**Height (cm)**	171.36 ± 0.11	171.09 ± 0.12	172.37 ± 0.24	< 0.001	157.91 ± 0.10	158.04 ± 0.10	156.02 ± 0.31	< 0.001
**Weight (kg)**	72.46 ± 0.19	71.15 ± 0.20	77.47 ± 0.45	< 0.001	58.21 ± 0.13	57.85 ± 0.12	63.41 ± 0.57	< 0.001
**BMI (kg/m** ^**2**^ **)**	24.62 ± 0.05	24.26 ± 0.05	26.00 ± 0.13	< 0.001	23.37 ± 0.05	23.18 ± 0.05	26.01 ± 0.21	< 0.001
**AST (U/L)**	25.30 ± 0.25	24.37 ± 0.27	28.87 ± 0.65	< 0.001	20.82 ± 0.11	20.51 ± 0.11	25.36 ± 0.60	< 0.001
**ALT (U/L)**	28.28 ± 0.33	26.34 ± 0.31	35.70 ± 0.99	< 0.001	17.67 ± 0.15	17.21 ± 0.15	24.16 ± 0.87	< 0.001
**BUN (mg/dL)**	14.98 ± 0.06	14.94 ± 0.07	15.17 ± 0.13	0.116	13.76 ± 0.06	13.56 ± 0.06	16.56 ± 0.27	< 0.001
**Cr (mg/dL)**	0.95 ± 0.002	0.94 ± 0.002	1.00 ± 0.004	< 0.001	0.70 ± 0.001	0.69 ± 0.001	0.81 ± 0.01	< 0.001
**eGFR (mL/min)**	95.15 ± 0.29	95.58 ± 0.31	93.53 ± 0.60	0.001	99.94 ± 0.28	100.87 ± 0.28	86.72 ± 1.21	< 0.001
**HTN, n (%)**	2,363 (36.6)	1,850 (35.5)	513 (41.3)	0.001	2,616 (29.6)	2,283 (27.8)	333 (55.1)	< 0.001
**DM, n (%)**	966 (15.0)	828 (15.9)	138 (11.1)	< 0.001	941 (10.4)	815 (9.9)	126 (20.9)	< 0.001
**Hyperlipidemia, n (%)**	1,289 (20.0)	1,013 (19.4)	276 (22.2)	0.068	2,151 (24.4)	1,938 (23.6)	213 (35.3)	< 0.001
**Smoking, n (%)**	2,126 (32.9)	1,681 (32.3)	445 (35.8)	0.327	419 (4.7)	374 (4.5)	45 (7.5)	0.027
**Alcohol, n (%)**	4,485 (69.5)	3,546 (68.0)	939 (75.5)	0.004	3,586 (40.6)	3,345 (40.7)	241 (39.9)	0.970
**Exercise, n (%)**	1,659 (25.7)	1,314 (25.2)	345 (27.8)	0.202	1,706 (19.3)	1,614 (19.6)	92 (15.2)	0.258
**Total intake (g)** Carbohydrate (g) Protein (g) Fat (g)	1843.67 ± 15.88 335.60 ± 2.14 86.40 ± 0.72 54.65 ± 0.66	1844.93 ± 17.06 339.55 ± 2.31 86.25 ± 0.76 53.99 ± 0.70	1838.82 ± 31.19 320.41 ± 4.36 87.01 ± 1.60 57.17 ± 1.44	0.854 < 0.001 0.655 0.040	1398.88 ± 10.32 263.85 ± 1.55 60.07 ± 0.45 38.17 ± 0.39	1405.74 ± 10.55 264.88 ± 1.60 60.29 ± 0.47 38.34 ± 0.39	1300.69 ± 30.84 249.17 ± 5.60 56.80 ± 1.32 35.80 ± 1.64	0.001 0.007 0.013 0.124
**Total fiber (g)** Cereals (g) Fruits (g) Vegetables (g)	27.07 ± 0.23 6.85 ± 0.09 3.74 ± 0.12 8.64 ± 0.09	27.63 ± 0.25 6.89 ± 0.10 3.95 ± 0.13 8.80 ± 0.10	24.89 ± 0.44 6.72 ± 0.20 2.92 ± 0.22 8.03 ± 0.17	< 0.001 0.452 < 0.001 < 0.001	23.31 ± 0.21 5.52 ± 0.07 4.82 ± 0.13 6.51 ± 0.07	23.44 ± 0.22 5.57 ± 0.07 4.86 ± 0.13 6.52 ± 0.07	21.45 ± 0.59 4.88 ± 0.19 4.30 ± 0.35 6.31 ± 0.23	0.001 0.001 0.106 0.348

BMI, body-mass index; AST, aspartate aminotransferase; ALT, alanine aminotransferase; BUN, blood urea nitrogen; Cr, creatinine; eGFR, estimated glomerular filtration rate; HTN, hypertension; DM, diabetes mellitus

### Associations between serum uric acids levels and dietary fiber intake

**Fig. 2:**
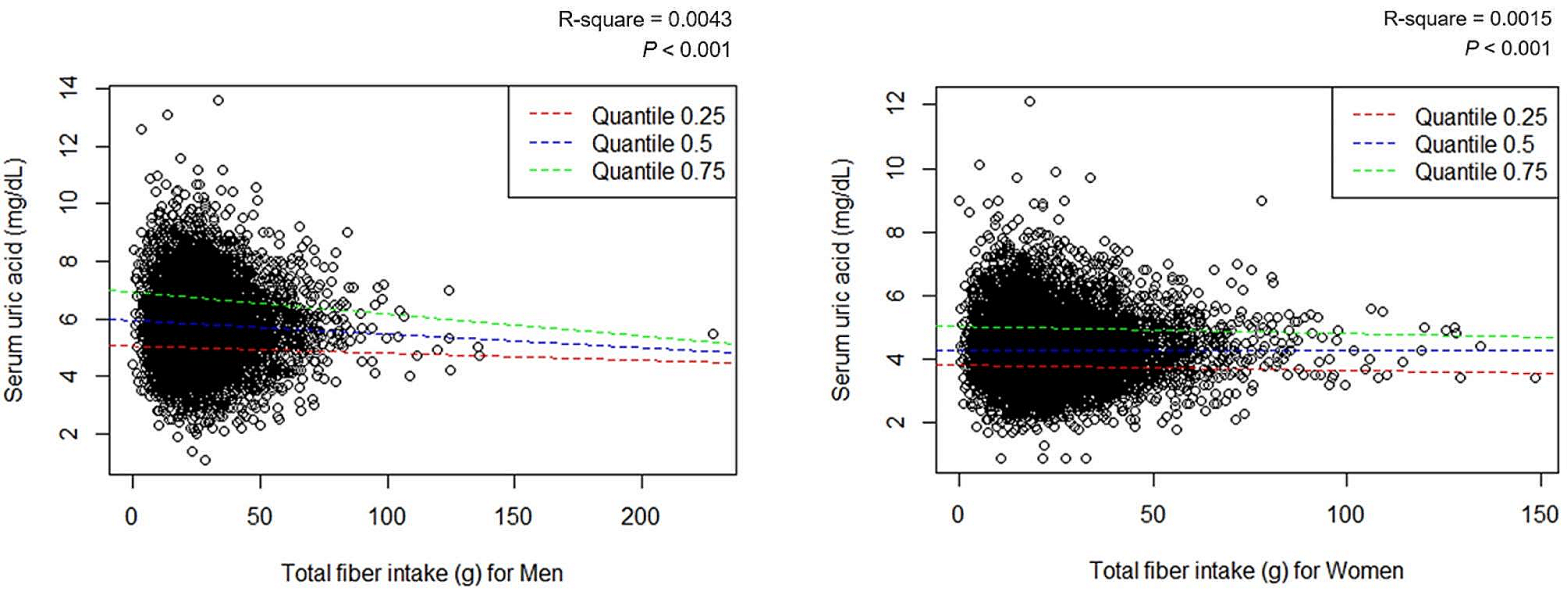
Scatter plot and the quantile regression plot exploring the association of serum uric acid levels with total fiber intake

When the continuous values ​​of serum uric acid and dietary fiber intake were evaluated by linear regression, there were significant associations in the crude model for both males and females in the total population (Fig. 2). The recommended dietary fiber intake was set based on the receiver operating characteristic (ROC) curve based on hyperuricemia status (Supplementary SF1). Consuming more than 27.9 g of dietary fiber in men and 20.7 g in women can reduce the risk of hyperuricemia by approximately 30% with odds ratios of 0.72 (0.62–0.83) and 0.71 (0.56–0.88) in men and women, respectively (Table 2).

**Table 2:** Odds ratios and 95% confidence intervlas for the hyperuricemia according to the optimal cut-off value of dietary fiber intake

Total fiber	Crude		Model 1		Model 2		Model 3	
	OR (95% CI)	*P*	OR (95% CI)	*P*	OR (95% CI)	*P*	OR (95% CI)	*P*
Men > 27.9 g	0.678 (0.587–0.784)	< 0.001	0.725 (0.624–0.841)	< 0.001	0.726 (0.625–0.845)	< 0.001	0.716 (0.615–0.834)	< 0.001
Women > 20.7 g	0.700 (0.575–0.853)	< 0.001	0.683 (0.557–0.838)	< 0.001	0.699 (0.564–0.866)	0.001	0.706 (0.563–0.884)	0.002

Reference group was set as a population taking dietary fiber below the optimal cut-off, and the odds ratios for taking sufficient dietary fiber were calculated by logistic regression analysis

Crude: unadjusted; Model 1: adjusted by age, body-mass index (BMI); Model 2: adjusted by age, BMI, alanine aminotransferase (ALT), glomerular filtration rate (GFR); Model 3: adjusted by age, BMI, ALT, GFR, hypertension, diabetes, hyperlipidemia, alcohol

OR, odds ratio; CI, confidence interval

### Risk of hyperuricemia according to the amount of dietary fiber intake

Dietary fiber was divided into quartiles. The odds ratio of hyperuricemia in the highest quartile group was evaluated by logistic regression using the lowest quartile as a reference (Table 3). High dietary fiber intake was found to significantly reduce the risk of hyperuricemia in both the total population and subgroups. The adjusted odds ratios (95% confidence interval) of the highest quartile corrected for the associated clinical variables were 0.61 (0.49–0.76) in the male population, and 0.65 (0.48–0.88) in the female population. We also evaluated the risk reduction according to the type of dietary fiber. Cereal fiber was significantly identified in both men and women, and fruit fiber was significant only in men. We attached additional tables for the results of each quartile (Supplementary Table ST1-2).

**Table 3:** Logistic regression analysis according to fiber types

Total fiber	Crude		Model 1		Model 2		Model 3	
	OR (95% CI)	*P*	OR (95% CI)	*P*	OR (95% CI)	*P*	OR (95% CI)	*P*
**Men**								
Total fiber	0.579 (0.474–0.706)	< 0.001	0.644 (0.522–0.793)	< 0.001	0.624 (0.505–0.770)	< 0.001	0.611 (0.493–0.757)	< 0.001
Cereal fiber	0.770 (0.635–0.934)	0.008	0.731 (0.600–0.890)	0.002	0.688 (0.563–0.841)	< 0.001	0.703 (0.572–0.863)	0.001
Vegetable fiber	0.728 (0.599–0.886)	0.002	0.777 (0.633–0.954)	0.016	0.795 (0.645–0.978)	0.030	0.761 (0.617–0.938)	0.011
Fruit fiber	0.606 (0.502–0.731)	< 0.001	0.724 (0.596–0.880)	0.001	0.691 (0.566–0.843)	< 0.001	0.716 (0.584–0.877)	0.001
**Women**								
Total fiber	0.671 (0.515–0.876)	0.001	0.613 (0.463–0.811)	0.001	0.641 (0.479–0.858)	0.003	0.653 (0.483–0.884)	0.006
Cereal fiber	0.669 (0.506–0.884)	0.005	0.680 (0.512–0.904)	0.008	0.685 (0.511–0.918)	0.011	0.721 (0.536–0.970)	0.031
Vegetable fiber	0.813 (0.630–1.049)	0.111	0.719 (0.549–0.941)	0.017	0.778 (0.587–1.030)	0.080	0.725 (0.541–0.971)	0.031
Fruit fiber	0.840 (0.637–1.108)	0.216	0.828 (0.621–1.104)	0.198	0.791 (0.586–1.066)	0.123	0.798 (0.584–1.090)	0.155

Reference group was the lowest quartile, and the odds ratios for the highest quartile were calculated by logistic regression analysis

Crude: unadjusted; Model 1: adjusted by age, body-mass index (BMI); Model 2: adjusted by age, BMI, alanine aminotransferase (ALT), glomerular filtration rate (GFR); Model 3: adjusted by age, BMI, ALT, GFR, hypertension, diabetes, hyperlipidemia, alcohol

OR, odds ratio; CI, confidence interval

### Subgroup analysis according to demographic variables

**Fig. 3:**
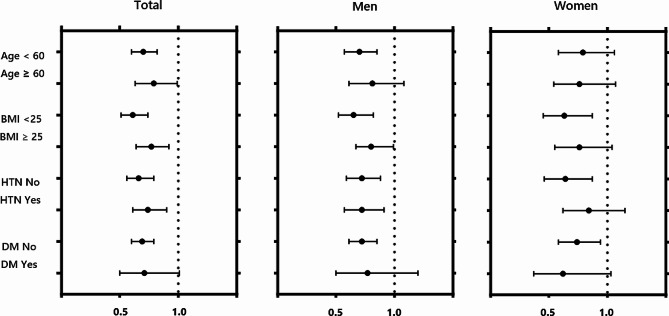
Subgroup analysis according to demographic variables. Reference group was set as a population taking dietary fiber below the optimal cut-off, and the odds ratios for taking sufficient dietary fiber were calculated by logistic regression analysis. Multivariable logistic regression model adjusted by age, body-mass index, alanine aminotransferase, glomerular filtration rate, hypertension, diabetes, hyperlipidemia, alcohol. OR, odds ratio; CI, confidence interval; BMI, body-mass index; DM, diabetes mellitus

In the subgroup analysis, the inverse association between dietary fiber and serum uric acid levels was particularly significant in young men with an adjusted odds ratio (95% confidence interval) of 0.93 (0.61–0.89) (*p* < 0.001). In addition, the association remained significant in people with normal weight with an adjusted odds ratio (95% confidence interval) of 0.61 (0.51–0.74) (*p* < 0.001). However, this inverse relationship was not statistically significant in diabetic patients (Fig.3).

## Discussion

This study was a nationwide cross-sectional study using KNHANES to investigate the association between serum uric acid level and dietary fiber intake. Among a total of 15,278 Korean adults (6,455 males/8,823 females), there was a significant inverse association between dietary fiber intake and serum uric acid level. In this study population, the prevalence of hyperuricemia among Korean adults with normal renal function was 12.1% in the total population, 19.3% in men and 6.8% in women. The serum uric acid levels were generally higher in men than in women, and it isconsistent with the findings from several epidemiologic studies [16, 17]. We thought that it isrelated to the role of sex hormones on hepatic metabolism or the renal and intestinal excretion of serum uric acid [18, 19].

When the recommended amount of daily fiber intake was estimated according to the hyperuricemia status using the optimal cut-off of the ROC curve, it was 27.9 g for men and 20.7 g for women. The average daily dietary fiber intake of Koreans was 23.2 g according to a previous study, which was higher than that of Americans (17.2 g) and Japanese (14.2 g) [20]. Considering the different dietary intake pattern by sex, the mean dietary fiber intake of the male population was higher at 24.8 g compared to that of female population at 21.6 g. However, intake per 1000 kcal was higher in women than it was in men (10.7 g in males and 12.6 g in females). This discrepancy is thought to be due to the higher consumption of alcohol and red meat in men compared to in women, which are foods that are calorically dense [15]. It is necessary to recommend increasing dietary fiber intake in Korean men with hyperuricemia, considering that the average dietary fiber intake is lower than the optimal amount calculated in this study.

The US and China nutritional survey study also showed an inverse association between total fiber and hyperuricemia [11, 12]. In the Korean survey, the food group that contributed most to dietary fiber intake was vegetables, and the combined dietary fiber intake from vegetables and cereals accounted for more than 50% of total intake (Supplementary Figure SF2). Our study, and those studies from the US and China have all shown an inverse correlation between cereal fiber intake and hyperuricemia. Because cereals are the main source of carbohydrate in most types of diet, they can help maintain the amount of dietary fiber intake[21]. These results suggest that intake of high-quality carbohydrates in cereals may prevent hyperuricemia. In addition, vegetable fiber intake can also lower the risk of hyperuricemia based on our results. In the US and Chinese studies, subgroup analyses of vegetable fiber did not confirm a significant relationship with hyperuricemia. Compared to the results of other countries, Korea reported a relatively higher intake of vegetable fiber (median daily value for vegetables: US = 2.80 g, China = 3.11 g, and Korea = 6.40 g). Therefore, we believe that this statistical association would have been easier to confirm statistically in Korean population with a higher intake of vegetable fiber.

Fruits are thought to worsen hyperuricemia, because the fructose that they contain is metabolized into uric acid. However, fruits also contain fiber and various nutrients (including vitamin C) that can lower uric acid; therefore, the effect of fruit consumption on hyperuricemia is complex [22]. In our study, a subgroup analysis focusing on fruit fiber components demonstrated a reduced risk of hyperuricemia in men, and no significant relationship in women (Table 3). The results of this study suggest that sufficient intake of all types of fiber may be helpful in lowering the risk of hyperuricemia. Based on this results, we suggest that strict fruit restriction may be unnecessary.

In the subgroup analysis, the inverse association between dietary fiber intake and serum uric acid levels was particularly significant in young men (Fig. 3). Serum uric acid levels are generally higher in men, and there is a tendency for serum uric acid levels to be higher in younger men than in older men [23]. Young men are considered at high risk for diseases associated with hyperuricemia; therefore, it is clinically meaningful that fiber intake may be helpful for these high-risk groups. In addition, the association between serum uric acid and dietary fiber intake in elderly people over 60 years old, obese populations with a BMI > 25 kg/m^2^, and populations with metabolic diseases are reported to be relatively weak (Fig. 3). In addition, intake of raw vegetables and fruits is limited due to the risk of hyperkalemia in patients with impaired renal function. Therefore, the results of this study are difficult to generalize to patients with metabolic diseases, including chronic kidney disease.

In fact, serum uric acid levels in pathological conditions are controversial, because there is an inverted U-shape association between blood pressure or blood sugar levels and serum uric acid levels [24, 25]. Additionally, in these high-risk populations, the effects of medications may be more significant than are the effects of dietary fiber intake [26]. For instance, the use of thiazide diuretics for hypertension can lead to an increase in uric acid levels. Conversely, losartan can decrease the uric acid levels. Among hyperlipidemia drugs, fibrate, which is used to lower triglycerides, is associated with a decrease in uric acid. The SGLT2 inhibitor class of diabetes drugs, which have been widely used recently, also reduces serum uric acid.

This study has the advantage of being a large-scale study that targets the nationwide population. However, only associations could be confirmed in this setting. For instance, although we attempted to correct for confounding variables, people with high dietary fiber intake may have had other lifestyles habits that decrease serum uric acid. Prospective studies using dietary fiber interventions may provide better evidence. In addition, since the nutrition survey method using the 24-hour recall method does not reflect individual dietary changes, it is possible that different results may be derived through improvement of the nutrition survey. Although it is implausible for a large population survey, a more comprehensive method such as food diary can be applied to a smaller patient group.

## Conclusions

Dietary fiber intake was inversely associated with the serum uric acid level, and this relationship was particularly significant in metabolically healthy young adults. Dietary fiber can inhibit the digestion and absorption of ingested purines, thereby reducing the production of serum uric acid, a metabolite of purines. In addition, dietary fiber may increase the excretion of uric acid from the body through the gastrointestinal tract, because it cleans the intestine and increases fecal excretion [27]. From a metabolic point of view, dietary fiber is a beneficial nutrient that lowers cholesterol and controls blood glucose levels [28, 29]. Improvements in other metabolic functions may have indirectly positive effects on the risk of hyperuricemia. Our results emphasize the importance of dietary fiber intake as a preventive or suppressive method for hyperuricemia and its associated medical conditions.

### Electronic supplementary material

Below is the link to the electronic supplementary material.

Supplementary Material 1

## Data Availability

The datasets generated and analyzed in this study are available in the KNHANES repository (https://knhanes.kdca.go.kr/knhanes/main.do).
