# Supplementary material for: Association between serum uric acid levels and dietary fiber intake in adults: the Korea national health and nutrition examination survey (KNHANES VII, 2016–2018)
Source: Nutr Metab (Lond). 2024 Jun 10;21:33. doi: 10.1186/s12986-024-00809-9 (PMC11165754; doi:10.1186/s12986-024-00809-9)
Supplement: Supplementary file 1 — Supplementary Material 1 [file 12986_2024_809_MOESM1_ESM.docx]

**Supplementary Table ST1.** Logistic regression analysis according to fiber types in men

| **Total fiber** | **Crude** | | **Model 1** | | **Model 2** | | **Model 3** | |
| --- | --- | --- | --- | --- | --- | --- | --- | --- |
|  | OR (95% CI) | *P* | OR (95% CI) | *P* | OR (95% CI) | *P* | OR (95% CI) | *P* |
| Total  Q2  Q3  Q4 | 0.782 (0.636-0.960)  0.681 (0.557-0.833)  0.579 (0.474-0.706) | 0.019  <0.001  <0.001 | 0.817 (0.655-1.019)  0.749 (0.609-0.923)  0.644 (0.522-0.793) | 0.073  0.007  <0.001 | 0.766 (0.610-0.961)  0.725 (0.587-0.896)  0.624 (0.505-0.770) | 0.021  0.003  <0.001 | 0.769 (0.607-0.974)  0.726 (0.583-0.905)  0.611 (0.493-0.757) | 0.030  0.004  <0.001 |
| Cereal  Q2  Q3  Q4 | 0.692 (0.566-0.846)  0.706 (0.582-0.857)  0.770 (0.635-0.934) | <0.001  <0.001  0.008 | 0.696 (0.568-0.852)  0.718 (0.590-0.874)  0.731 (0.600-0.890) | 0.001  0.001  0.002 | 0.679 (0.550-0.838)  0.695 (0.569-0.849)  0.688 (0.563-0.841) | <0.001  <0.001  <0.001 | 0.688 (0.556-0.851)  0.720 (0.585-0.886)  0.703 (0.572-0.863) | 0.001  0.002  0.001 |
| Vegetable  Q2  Q3  Q4 | 0.834 (0.681-1.021)  0.775 (0.634-0.947)  0.728 (0.599-0.886) | 0.079  0.013  0.002 | 0.867 (0.703-1.071)  0.829 (0.674-1.020)  0.777 (0.633-0.954) | 0.185  0.077  0.016 | 0.861 (0.694-1.067)  0.810 (0.655-1.003)  0.795 (0.645-0.978) | 0.171  0.053  0.030 | 0.871 (0.702-1.080)  0.772 (0.621-0.960)  0.761 (0.617-0.938) | 0.207  0.020  0.011 |
| Fruit  Q2  Q3  Q4 | 1.194 (0.943-1.511)  0.868 (0.722-1.044)  0.606 (0.502-0.731) | 0.140  0.133  <0.001 | 1.115 (0.877-1.417)  0.939 (0.777-1.135)  0.724 (0.596-0.880) | 0.372  0.515  0.001 | 1.101 (0.859-1.410)  0.884 (0.723-1.080)  0.691 (0.566-0.843) | 0.447  0.226  <0.001 | 1.052 (0.815-1.358)  0.899 (0.733-1.103)  0.716 (0.584-0.877) | 0.697  0.306  0.001 |

OR, odds ratio; CI, confidence interval. Reference group was the lowest quartile group (Q1).

Quartiles for men: Total fiber (Q1< 17.05, 17.05 ≤Q2< 24.69, 24.69 ≤Q3< 34.18, 34.18 ≤Q4); Grain fiber (Q1< 3.18, 3.18 ≤Q2< 5.20, 5.20 ≤Q3< 8.58, 8.58 ≤Q4); Vegetable fiber (Q1< 4.50, 4.50 ≤Q2< 7.61, 7.61 ≤ Q3< 11.26, 11.26 ≤Q4); Fruit fiber (Q1= 0, 0< Q2< 0.30, 0.30 ≤Q3< 4.96, 4.96 ≤Q4)

Crude: unadjusted; Model 1: adjusted by age, body-mass index (BMI); Model 2: adjusted by age, BMI, alanine aminotransferase (ALT), glomerular filtration rate (GFR); Model 3: adjusted by age, BMI, ALT, GFR, hypertension, diabetes, hyperlipidemia, alcohol

**Supplementary Table ST2.** Logistic regression analysis according to fiber types in women

| **Total fiber** | **Crude** | | **Model 1** | | **Model 2** | | **Model 3** | |
| --- | --- | --- | --- | --- | --- | --- | --- | --- |
|  | OR (95% CI) | *P* | OR (95% CI) | *P* | OR (95% CI) | *P* | OR (95% CI) | *P* |
| Total  Q2  Q3  Q4 | 0.801 (0.622-1.032)  0.621 (0.475-0.811)  0.671 (0.515-0.876) | 0.087  0.001  0.001 | 0.748 (0.573-0.978)  0.603 (0.456-0.796)  0.613 (0.463-0.811) | 0.034  <0.001  0.001 | 0.721 (0.544-0.956)  0.584 (0.438-0.778)  0.641 (0.479-0.858) | 0.023  <0.001  0.003 | 0.749 (0.561-1.001)  0.604 (0.449-0.814)  0.653 (0.483-0.884) | 0.051  0.001  0.006 |
| Cereal  Q2  Q3  Q4 | 0.821 (0.634-1.063)  0.778 (0.597-1.014)  0.669 (0.506-0.884) | 0.134  0.064  0.005 | 0.788 (0.605-1.026)  0.792 (0.603-1.041)  0.680 (0.512-0.904) | 0.077  0.094  0.008 | 0.771 (0.586-1.014)  0.780 (0.587-1.036)  0.685 (0.511-0.918) | 0.063  0.086  0.011 | 0.763 (0.577-1.011)  0.798 (0.598-1.065)  0.721 (0.536-0.970) | 0.059  0.126  0.031 |
| Vegetable  Q2  Q3  Q4 | 0.748 (0.568-0.985)  0.853 (0.663-1.097)  0.813 (0.630-1.049) | 0.039  0.215  0.111 | 0.692 (0.520-0.920)  0.840 (0.648-1.090)  0.719 (0.549-0.941) | 0.012  0.189  0.017 | 0.724 (0.540-0.971)  0.921 (0.699-1.212)  0.778 (0.587-1.030) | 0.031  0.555  0.080 | 0.711 (0.524-0.963)  0.848 (0.641-1.122)  0.725 (0.541-0.971) | 0.028  0.248  0.031 |
| Fruit  Q2  Q3  Q4 | 1.041 (0.801-1.354)  0.892 (0.684-1.165)  0.840 (0.637-1.108) | 0.763  0.401  0.216 | 1.083 (0.827-1.419)  0.943 (0.711-1.250)  0.828 (0.621-1.104) | 0.560  0.683  0.198 | 1.025 (0.772-1.362)  0.877 (0.654-1.177)  0.791 (0.586-1.066) | 0.862  0.381  0.123 | 0.993 (0.740-1.332)  0.932 (0.691-1.258)  0.798 (0.584-1.090) | 0.963  0.646  0.155 |

OR, odds ratio; CI, confidence interval. Reference group was the lowest quartile group (Q1).

Quartiles for women: Total fiber (Q1< 13.94, 13.94 ≤Q2< 20.34, 20.34 ≤Q3< 29.33, 29.33 ≤Q4); Grain fiber (Q1 < 2.54, 2.54 ≤Q2< 4.24, 4.24 ≤Q3< 6.76, 6.76 ≤Q4); Vegetable fiber (Q1< 3.16, 3.16 ≤Q2< 5.41, 5.41 ≤Q3< 8.56, 8.56 ≤Q4); Fruit fiber (Q1= 0, 0< Q2< 2.04, 2.04 ≤Q3< 6.64, 6.64 ≤Q4)

Crude: unadjusted; Model 1: adjusted by age, body-mass index (BMI); Model 2: adjusted by age, BMI, alanine aminotransferase (ALT), glomerular filtration rate (GFR); Model 3: adjusted by age, BMI, ALT, GFR, hypertension, diabetes, hyperlipidemia, alcohol

**Supplementary Figure SF1.** Optimal cut-off for dietary fiber intake calculated by ROC curves


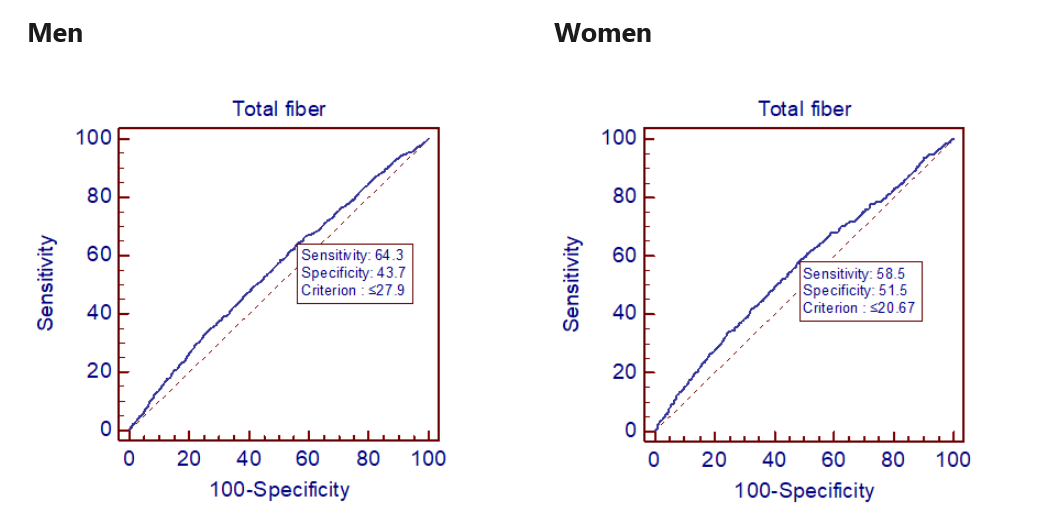


**Supplementary Figure SF2.** Dietary intake status of the Korean population from 2016 to 2018


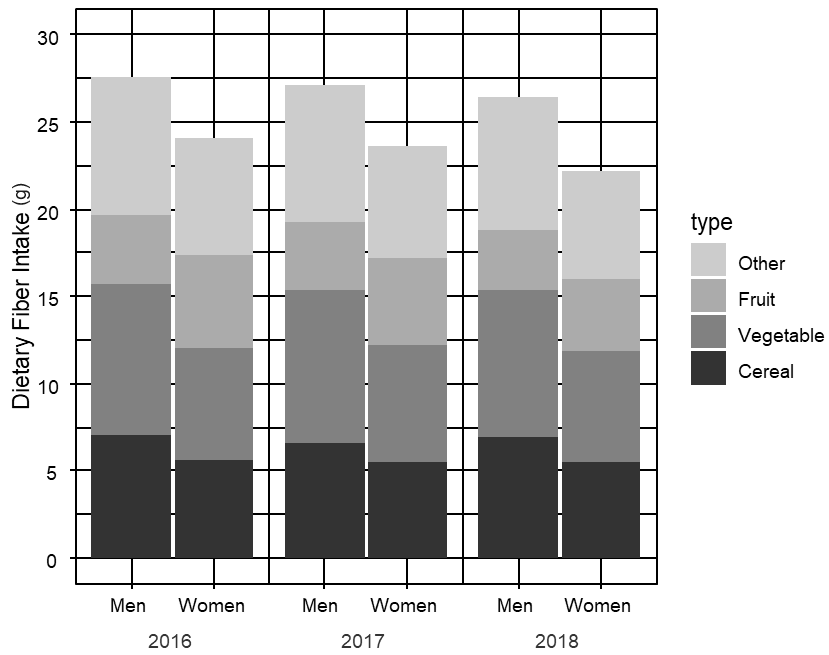


Sources of dietary fiber categorized as ‘Other’ are a variety of food groups, including legumes, seaweeds, starches, mushrooms and nuts.
